# “Web impingement” of the ankle: a case report

**DOI:** 10.1007/s00167-012-2077-4

**Published:** 2012-06-12

**Authors:** Kars P. Valkering, Pau Golanó, C. Niek van Dijk, Gino M. M. J. Kerkhoffs

**Affiliations:** 1 Department of Orthopaedic Surgery, Academic Medical Center, University of Amsterdam, PO Box 22700, 1100 DE Amsterdam, The Netherlands; 2 Laboratory of Arthroscopic and Surgical Anatomy, Department of Pathology and Experimental Therapeutics (Human Anatomy Unit), University of Barcelona, c/Feixa Llarga s/n (Campus Bellvitge), L’Hospitalet de Llobregat, 08907 Barcelona, Spain

**Keywords:** Ankle arthroscopy, Anterior ankle impingement, Soft-tissue impingement, Fibrous band

## Abstract

This case report presents two patients with persisting anterior ankle impingement pain after an ankle distortion. A web-like intra-articular fibrous band was discovered and resected. The patients presented were, after a 1-year follow-up, pain free.

*Level of evidence* IV.

## Introduction

Anterior ankle pain is a common complaint after an ankle sprain. Most patients are successfully treated non-operatively. However, some patients remain symptomatic. Both Biedert [1] and Takao et al. [11] showed the importance of ankle arthroscopy in cases where the cause of residual pain after an ankle sprain was, after clinical examination and complementary investigations, undiagnosable. Biedert [1] described several conditions showing good results after arthroscopic interventions in two-thirds of all patients. One-third showed unsatisfactory results mainly because of degenerative changes. In this article, two cases of undiagnosable posttraumatic anterior ankle impingement pain are described. Both were caused by a seldom seen intra-articular fibrous band. Both cases are illustrated using arthroscopy images and an anatomical specimen.

## Case report

Two patients with ankle complaints were referred to our out-patient clinic with identical symptoms. A 21-year-old male (patient 1) reported a right ankle sprain one year before, and a 38-year-old male (patient 2) sustained an identical trauma two years before to his left ankle. Sports activities (jumping, running, soccer) were restricted due to pain on the anteromedial side of the ankle joint. No restrictions were reported for ADL. At physical examination, the ankles showed a full range of motion and were ligamentous stable. A recognizable superficial ankle pain could be provoked by compressing the anteromedial side of the ankle joint. Standard anteroposterior and lateral radiographs and a complementary oblique anteromedial impingement (AMI) radiograph [14] of the ankle displayed no abnormalities.

Magnetic resonance imaging (MRI) evaluation was performed 3 months (patient 1) and 12 months (patient 2) post trauma. In both cases, no particular bony, ligamentous or soft-tissue pathology could be diagnosed. The only apparent finding was a mild increase in intra-articular fluid.

A standard anterior ankle arthroscopy was performed, and although MRI evaluation was not conclusive, soft-tissue impingement was suspected.

**Fig. 1:**
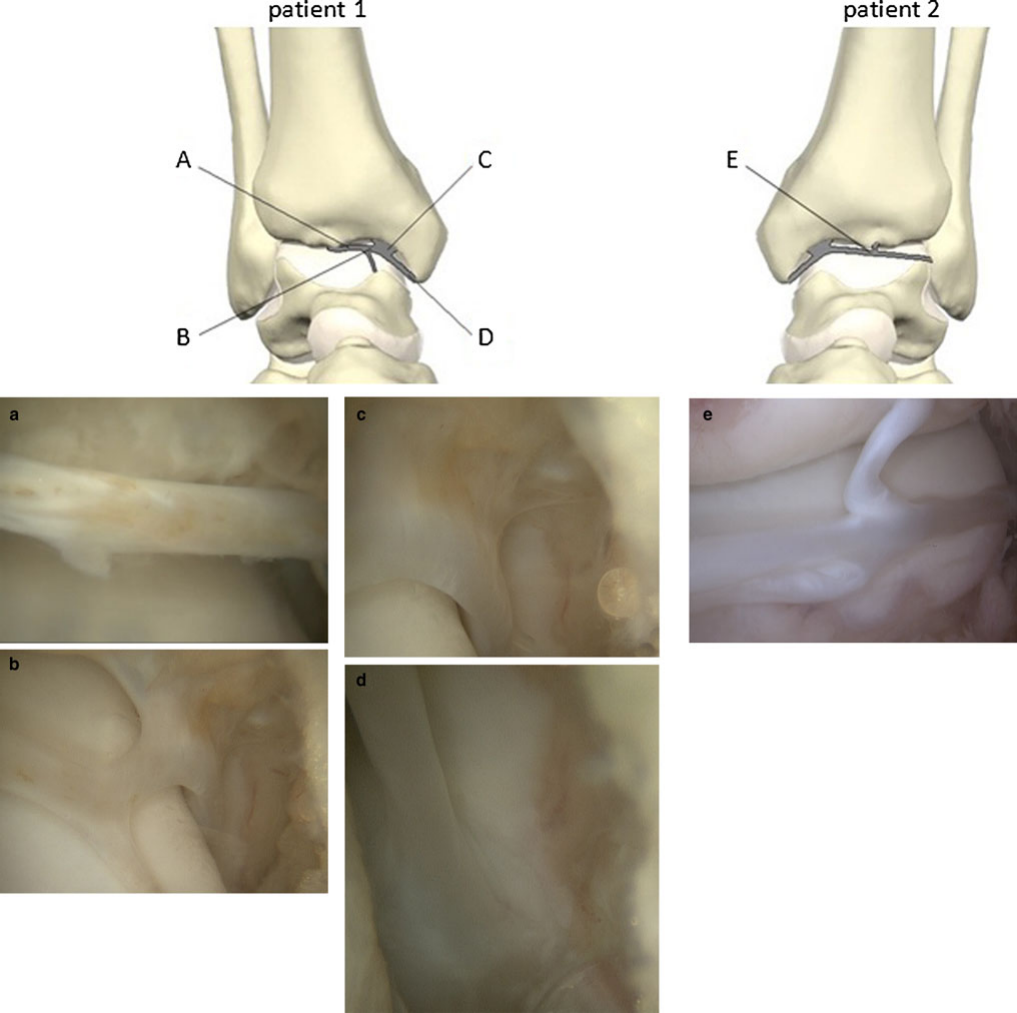
Intra-articular fibrous bands creating a web over the anteromedial talus with its attachments to the medial malleolus, distal tibia rim and talus

**Fig. 2:**
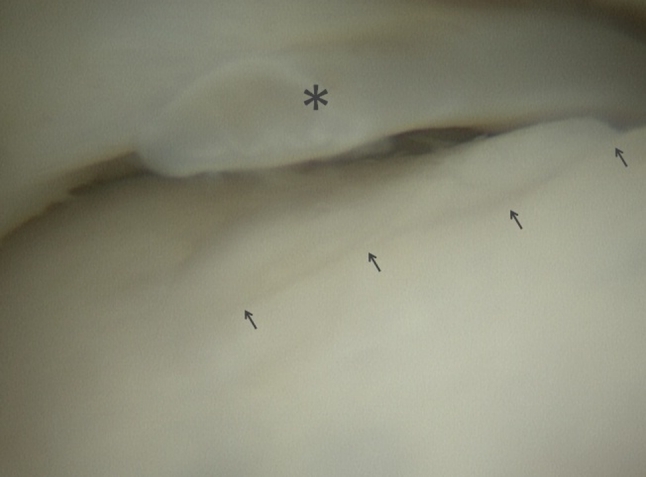
Detail of the anteromedial talus with a chondral lesion illustrated by *arrows*. The *asterisk* indicates a partially resected fibrous band

During the operation, a web-like intra-articular fibrous band was discovered in both cases, extending from the medial malleolus to the anterior distal tibia rim and talus, creating a web over the anteromedial portion of the talus (Fig. 1a–e). After the removal of the band, a superficial fraying of the talus (a sign of the impingement of the band on the talar surface) was noticed in patient 1 (Fig. 2). At a 1-year follow-up, both patients had no swelling, a pain-free full range of motion and a maximum AOFAS score.

Histology of the fibrous band showed fibrous tissue with broad collagen and normal fibroblasts resembling ligamentous tissue.

## Discussion

The most important findings of the present study were the discovery of an intra-articular fibrous web over the anteromedial talus causing anterior ankle impingement and the value of ankle arthroscopy both as a diagnostic tool and as a treatment modality.

A complication of an ankle trauma is the development of anterior ankle impingement. It can be classified according to its location (anterolateral, anterocentral, anteromedial) and to its biological substrate causing the impingement; most commonly: post-traumatic synovitis, capsular scarring or bony osteophytes [5]. Bony osteophytes may lead to entrapment of the adjacent anterior joint capsule and give rise to the actual pain sensation [12]. Soft-tissue pathology can also cause anterior ankle impingement in the absence of underlying osteophytes. Excellent functional outcomes after arthroscopic resection of anteromedial ankle impingement were reported by Murawski and Kennedy [8].

Transarticular fibrous bands are distinct band-like structures which are firmly attached to capsule, articular surface or bone. Johnson was the first to describe transarticular fibrous bands [6]. Guhl attributes the origin of these bands to a cellular reaction following an intra-articular hematoma [4]. An explanation for the fibrous bands occurring more frequently in the anterior recess of the ankle is the larger volume of the anterior recess compared to the posterior recess, allowing more blood to accumulate after a trauma. Because of the frequent association of fibrous bands with fractures, a careful inventory of the ankle joint should be performed if a fibrous band is encountered during an ankle arthroscopy [10]. Ankle fractures are known to cause arthrofibrosis with its negative effect on joint function [13]. Considering the etiology of intra-articular fibrous bands, the consequence of finding one should prompt a careful inspection of the ankle joint for occult pathology [1, 9, 10].

Fibrous bands differ from meniscoid tissue. The main distinction being that the meniscoid is attached at one end and the fibrous band is attached at both ends. The meniscoid is flattened and usually tapering at its free end and has its origin at the inferolateral gutter on the anterior talofibular ligament [2, 10] or on the deltoid ligament on the medial side [3, 7]. A fibrous band is cord-like in cross section and can be found anywhere in the joint [10].

**Fig. 3:**
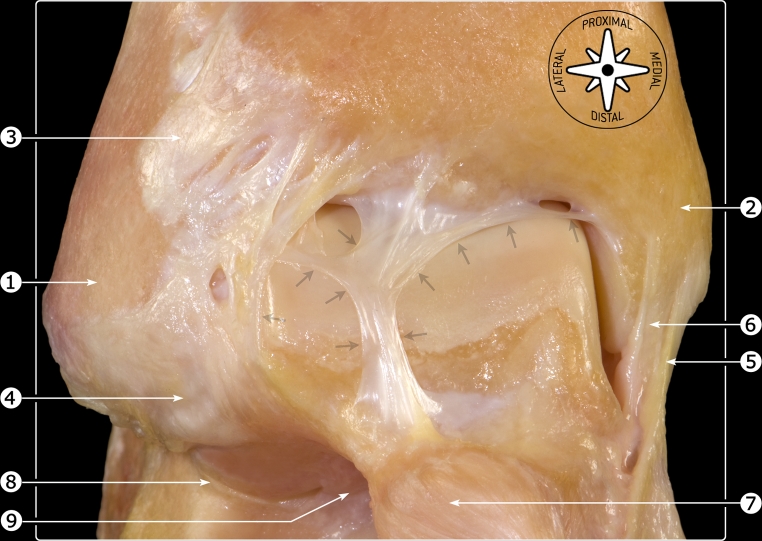
Anatomical ankle specimen. The arrows indicate a fibrous band extending from the distal tibia to the talus and fibula. lateral malleolus (*1*), medial malleolus (*2*), anterior tibiofibular ligament (*3*), anterior talofibular ligament (*4*), tibionavicular part of deltoid ligament (*5*), anterior tibiotalar part of deltoid ligament (*6*), dorsal talonavicular ligament (*7*), subtalar joint (*8*), tarsal sinus (*9*)

A specimen was prepared by Golanó in order to illustrate fibrous bands in the anterior ankle recess causing a web over the anterior dome of the talus (Fig. 3). Following repetitive impingement of the talar surface on the web with active dorsiflexion, a superficial fraying of the cartilage of the anterior dome of the talus can be found (Fig. 2). This is in accordance with Guhl and Stones findings [4].

Limitations of the present study are the small patient group and short follow-up. Tol et al. [12] showed excellent or good results after a 5–8-year follow-up for the arthroscopic treatment of anterior soft-tissue ankle impingement. Comparable long-term results for the treatment of the pathology of the present study are lacking.

MRI is valuable in the diagnostic workup of ankle impingement lesions [5]. In cases of fibrous bands of the ankle, MRI is less sensitive. However, the bands are most evident on axial and sagittal proton-density images [5, 9]. In both patients, MRI evaluation was negative for the intraoperatively diagnosed fibrous band. In our opinion, it is the clinical suspicion for soft-tissue impingement that sets the indication for anterior ankle arthroscopy.

## Conclusion

An ankle distortion can result in the formation of intra-articular fibrous bands causing “web impingement” of the ankle. Anterior ankle arthroscopy is of great value in the diagnostic work-up and treatment. If intra-articular fibrous bands are encountered, inspection of the ankle for the coexistence of occult pathology is indicated. In the present study, arthroscopy was superior to MRI in detecting this type of pathology.
